# Diamel Therapy in Polycystic Ovary Syndrome Reduces Hyperinsulinaemia, Insulin Resistance, and Hyperandrogenaemia

**DOI:** 10.1155/2012/382719

**Published:** 2012-06-21

**Authors:** Arturo Hernández-Yero, Felipe Santana Pérez, Gisel Ovies Carballo, Eduardo Cabrera-Rode

**Affiliations:** Department of Methodology of the Investigation, National Institute of Endocrinology, Havana, MD. Apdo.6275, Habana 6, Cuba

## Abstract

For to determine the effect of Diamel on the insulin resistance, insulin sensitivity, and sexual hormones results in women with polycystic ovary syndrome (PCOS). A study was carried out on 37 patients with this disorder. A triple-blind clinical trial was designed in which the Diamel food supplement was compared with a placebo. The women with reproductive ages were randomly distributed in two groups, with 18 and 19 women respectively, and they took Diamel or placebo and were followed up during 6 months with clinical and biochemical evaluation. A significant decrease in the HOMA-IR from the initial value at six months was observed in the group with Diamel. The insulin sensitivity improved considerably in this group. The rate of menstrual recovery was higher in the group with Diamel, and two patients from this group obtained pregnancy. The hormone levels shows a significant decrease in testosterone at 3 months in the group with Diamel compared with the control group. The LH also decreases in the same group when comparing the start with 6 months.We concluded that the Diamel decreases insulin resistance and improves sensitivity to this hormone in women with PCOS, with improvement in the levels of LH and testosterone.

## 1. Introduction

Polycystic ovary syndrome (PCOS) is a heterogeneous disorder characterized by chronic anovulation and the cutaneous effects of hyperandrogenism [1–3].

There is a high prevalence of PCOS in women with type 2 diabetes mellitus, and there is evidence that insulin resistance and associated compensatory hyperinsulinaemia play a central role in the pathogenesis of PCOS in some women [4–7]. The metabolic aspects of this syndrome include insulin resistance, obesity, lipid abnormalities, and an increased risk for impaired glucose tolerance and type 2 diabetes mellitus [7–9].

Hyperinsulinaemia and insulin resistance are more common among women with PCOS than women without the condition regardless of whether they are obese or not [1, 8]. High insulin levels can increase the amount of androgens produced by ovarian theca cells in synergism with LH. It has been suggested that hyperandrogenism and insulin resistance occur early on in life and might even originate during foetal life and detected clinically before puberty [9, 10]. A potential mechanism for insulin resistance in PCOS women increased serine phosphorylation of the insulin receptors [8]. Serine phosphorylation of IRS-1 (insulin receptor substrate-1) has been suggested as a mechanism for TNF-*α*-mediated insulin resistance and modulated the activity of the key regulatory enzyme of androgen biosynthesis, P450c17 in the adrenal and ovarian steroidogenic tissues [4, 6, 8].

 The high prevalence of PCOS observed in certain ethnic groups also seems to be identified with a high risk of insulin resistance [8], and certain drugs used to improve insulin sensitivity have been included in the treatment for these women [6, 7, 11]. These drugs include metformin and the glitazones, which have had good results in controlling insulin resistance in patients with the syndrome [12–16]. New studies would be of utility for research into new drugs to increase another therapeutical possibilities. Diamel is a well-known product as a vitamin supplement used to treat type 2 diabetes mellitus. It can improve insulin sensitivity and the *β*-cell function in people suffering from this disease [17]. It could be an alternative therapy for women with PCOS if it can be proved that it is capable of decreasing insulin resistance in these patients and improve the performance of insulin produced by pancreatic cells. We decided to conduct research on women diagnosed with PCOS and evaluate the possible effect that this supplement has on the insulin sensitivity index, and to find out how the gonadotropins and testosterone levels in these patients react to the treatment.

The studies carried out on the experimental and preclinical models showed that this vitamin supplement improves glucose tolerance, and it does not contain any toxic elements [17, 18]. The product is approved by the National Institute of Nutrition and Food Safety and although earlier tests with Diamel have not been carried out on patients with PCOS, favourable results in diabetics with insulin resistance have been obtained in which a decrease in the triglyceride levels was observed along with an increase in HDL-cholesterol and an improvement of HOMA-IR and HOMA-B indexes in a 6-month period [17]. This implies that it could have a favourable response in women with PCOS and insulin resistance.

Diamel contains trace elements, amino acids, vitamins, lettuce extract, and cranberry extract, which are activated by means of a molecular magnetization process. The laboratory researchers who produce it state that it is designed to stimulate the pancreatic metabolism with its natural activated components that work like biocatalysts. The secret to its effectiveness lies in molecular activation process of the natural ingredients that it contains. Moreover, if we take into account the fact that the stability of human organic systems is progressively deteriorated due to the harmful chemical reactions that cause this cellular oxidative stress, antioxidants that it contains could prevent or delay such reactions. After the molecular activation process developed by the Catalysis laboratories, a biophysical analysis was carried out on the electric conductivity and the thermal stability of different products, including Diamel, whose molecules and components were seen to be powerful reagents [17, 18]. 

It is worthwhile mentioning the fact that even though no previous studies have been published about the Diamel and PCOS, but some gynaecologists from Kuwait have used this supplement on patients with PCOS, and they have observed an improvement in their menstrual disorders with ovulation recovery (personal data). Therefore, this paper would be the first documented study to provide results to actually consider using the supplement. 

We aimed at identifying the response of women with the PCOS in treatment with Diamel for to evaluate insulin sensitivity and sexual hormones results.

## 2. Patients and Methods

The patients were recruited from ambulatory care in National Institute of Endocrinology. Vaginal ultrasound was performed in all subjects, and polycystic ovaries were diagnosed if ovaries were enlarged with more than 10–12 small antral follicles of 2–8 mm diameter with the typical cortical arrangement. 

 One hundred four women with PCOS were recruited consecutively during two years, and those that presented HOMA-IR index greater than 2.6 were included in the investigation.A Phase III triple*-*blind, placebo-controlled, random clinical trial was carried out with placebo that had similar characteristics as those of Diamel. The study lasted 6 months starting from the screening of the patients.

- 60 boxes of vials were handed out, numbered 1 to 60, according to the random number chart. One separate vial was assigned to each patient (30 of which correspond to Diamel and 30 to the placebo).
- Neither the researchers nor the patients knew whether the A or B vials contained the Diamel or the placebo capsules.
- At the end of the 6-month follow up and so as to statistically process the results, the first sealed envelope was opened. It contained information about the random distribution of the corresponding vial numbers for group A and group B.
- The identification of these vials was kept secret in another second-sealed envelope that was kept by an intermediary who was not involved in the research programme. This was handed over once the study with the statistical process had been completed to identify the vials.
- The envelopes and the administration of the Diamel or placebo were monitored by a nurse who was not directly involved in the study. This person was in charge of giving the researchers the corresponding envelope for each patient included in the trial. Until all the statistics had been processed, the link between the groups A and B and the placebo and Diamel were not revealed.
- *Diagnostic Criteria. *Patients who had been diagnosed with PCOS according to the Rotterdam criteria were included [19].

### 2.1. Inclusion Criteria 

Including criteria are the following:

- women in the reproductive stage (between 18 and 35 years old) who have been diagnosed with PCOS according to Rotterdam criteria,
- HOMA-IR greater than 2.6, and
- women who have not been treated by PCOS beforehand.

### 2.2. Exclusion Criteria

Excluding criteria are the following:

- patients with type 2 diabetes mellitus,
- patients with Cushing's syndrome,
- hyperprolactinemia,
- congenital adrenal hyperplasia due to a 21-hydroxylase deficiency,
- virilizing ovarian tumor,
- pregnancy, and
- ovarian cysts that are bigger than 10 mm.

### 2.3. Criteria to Leave the Study

They are the follows:

- serious adverse effects related to the supplement,
- patients who refused to carry on with the study and decided to leave,
- development of some of the processes or entities that are established in the exclusion criteria, and
- pregnancy (this will be recorded as being a favourable response to the supplement).

### 2.4. Treatment

Members of both groups were trained about the change of their life styles on their diet, and an increase in physical activity through walks or supervised exercises at least 5 times a week was recommended.

### 2.5. Dosage Used

The maximum maintenance dose of Diamel was 3960 mg (6 capsules) divided out into 2 capsules before breakfast, midmorning snack, and lunch were prescribed, based on the previous experience of the recommended therapeutic dosage. (See Table 1 with the components of Diamel).

### 2.6. Ultrasound

Before the study began, to each patient was made a transvaginal ultrasound in order to need anatomical characteristics the ovaries.

### 2.7. Biochemical, Hormone Analysis, and Indexes Used

Fasting blood samples were drawn during the morning in follicular phase (cycle days 3–5); patients with cycle length >3 months had the blood samples drawn on a random cycle day. All the laboratory measurements and indexes were recorded at the beginning, at 3 months and at 6 months of the follow up, as follows

- fasting blood glucose levels,
- fasting insulin levels,
- HOMA-IR index,
- Raynaud index,
- FSH and LH,
- Testosterone,lipids (cholesterol, Triglycerides, HDL-Cholesterol and LDL-Cholesterol),
- prolactin, and
- postinhibition of basal cortisol with 1 mg of dexamethasone.

At start study (point 0), at 3 months and at 6 months, blood glucose and insulin levels were taken in the morning, then after approximately 10–12 hours of fasting, three samples were taken every 5 minutes to measure the HOMA-IR, established by Wallace and colleagues [20, 21]. Moreover, Raynaud's index was used to determine insulin sensitivity [22, 23].

HOMA-IR: insulin resistance index:

(1) $$\begin{array}{cc} & \text{HOMA-IR}\\ & =\frac{\text{Fasting insulinemia}\;\left(\mu \text{U}/\text{mL}\right)\times \text{fasting glucose}\;\left(\text{mmol}/\text{L}\right)}{22.5}. \end{array}$$

It is considered to have increased when it is above 2.6.

Raynaud: insulin sensitivity index:

(2) $$\begin{array}{c} \text{Raynaud}=\frac{40}{\text{Fasting Insulinemia}\;\left(\mu \text{U}/\text{mL}\right)\;}. \end{array}$$

Normal sensitivity was considered to be at the cut-off point equal or less than 0.33.

### 2.8. Statistical Analysis and Ethical Aspects

The results were shown as means ± SD, and two-tailed Fisher's exact test was used to determine differences between groups. For menstrual pattern data and HOMA-IR and Raynaud indexes results comparisons within and between groups, nonparametric test (Kruskal-Wallis H. test and the Mann-Whitney *U* test) were used. Nonparametric tests were also used to examine treatment effects at start, 3 months and 6 months (within and between groups). *P* < 0.05 was considered significant for all tests.

 Patients' consent. An informed consent form was filled in by each patient. This form outlines the objectives of clinical trial and details of such. By signing the form, the subject expresses their willingness and agrees to take part in this research programme.

## 3. Results

**Figure 1:**
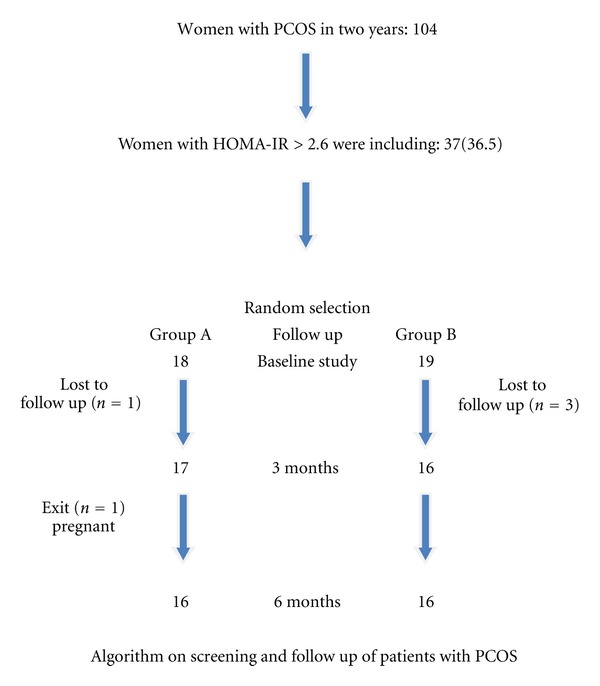


Figure 1 is a data-flow diagram on screening and subsequent follow up of the patients; we took two years to find 104 women with PCOS. 36.5% of patients have insulin resistance according to the HOMA-IR index. Two patients from group A (Diamel) and another 3 from group B (placebo) left the study by different causes.

Figure 1 shows the patient screening process over two years in which 36.5% of the 104 patients with PCOS were found to meet the criteria established on the existence of insulin resistance; 16 patients in each group completed the follow up stage up to the ending of study.

Table 2 contains the clinical features of the patients with PCOS. Obesity is predominant in both groups, and there are not any significant differences in the features assessed.

Table 3 contains the baseline values of the lipid and hormone measurements taken at the start of study. No significant differences were observed.

Table 4 contains the patients' physical evolution shown in weight and body mass index (BMI). Although there was a slight gradual decrease in weight in both groups, there were no significant differences.

On studying the evolution of the HOMA-IR index (Table 5), a significant drop from the initial value to the value given at 6 months is observed in the group that is administered Diamel. Meanwhile the decrease in this index in the group administered placebo is much smaller, even insignificant.

Table 6 contains data on the evolution of the insulin sensitivity by Raynaud index, which increases considerably especially in the group given Diamel.

Table 7 shows the changes in the irregular menstrual pattern in both groups. More patients in the group with Diamel start having normal periods again, compared to those in the control group, and two of the patients from the former group even manage to conceive: one of them obviously leaves the study for this reason at 3 months and the other patient conceives two weeks after the study has finished. The first woman has a miscarriage at 12 weeks. 

Table 8 shows the evolution of hormone levels. There is a significant decrease in testosterone at 3 months in the group treated with Diamel compared to the group that is given placebo. The LH also drops in the former group from the start at 6-month point; although it was smaller in this group than the used placebo, these differences did not reach statistical signification.

## 4. Discussion

The use of drugs such as metformin, which improves insulin sensitivity and decreases resistance to this hormone, has had good results in some studies with the decrease of high insulin levels and improvement of hyperandrogenism [14, 16]. Some studies have suggested a lower miscarriage rates if metformin treatment is continued in the first trimester, but this remains unresolved. There is less risk of miscarriage in women using this drug [24, 25]. However, there are not many other alternative drug treatments to reduce or prevent the signs and symptoms caused by PCOS; for this reason, we decided to study the use of the food supplement called Diamel. It has already proved to be effective in patients with type 2 diabetes mellitus; it does not have any adverse effects [17, 18], and it could be another alternative for women suffering from the syndrome if it proves to be capable of reducing insulin resistance. The components in Diamel are activated by means of electromagnetic procedures that can enhance the metabolic changes in patients, and the lettuce extract reduces the amount of glucose absorbed during the digestion process. The cranberry extract contains anthocyanosides and tannins which improve the microcirculation and the secretion of insulin. Cranberries and blueberries have proved to be beneficial for both men and women as it enhances insulin sensitivity and reduces resistance to this hormone [26]. 

Our research work involved two years of screening patients with PCOS and insulin resistance which was identified by the HOMA-IR index, as shown in the follow up algorithm (Figure 1). Only 36.5% of 104 patients that were initially studied satisfied the criteria. Even though the proposed figure of 60 women with PCOS, and a HOMA-IR index >2.6 was not reached, these circumstances limit the reach and the results of our investigation, despite we considered of utility in present to these data, since it is a first study that analyzes the possible influence of Diamel in a sample of women with PCOS and we were able to make an acceptable evaluation with the 37 patients who did satisfy aforesaid criteria. Other investigations with a greater sample of patients are necessary to validate these results.

None of the patients left the study during the follow up stage due to adverse effects caused by the food supplement. Indeed insulin sensitivity progressed favourably, and there was a significant decrease in the resistance to the hormone in the group being treated with Diamel. We found diminution of the insulin resistance in the group with the supplement, when compared the beginning with month 6 although statistical meaning is not appraised when it is compared with the placebo group; the decrease in the insulin resistance was not significance in this control group, and this is thought to have been due to a slight weight loss and drop in the BMI. Changing the life style is a proven first-line treatment for the majority of overweight women with PCOS. Excessive weight gain must be avoided. There are many clinical benefits in reducing the body weight by 5–10%. It improves the psychological results, the reproductive conditions (menstrual cycle, ovulation, and fertility), and the metabolic conditions, insulin resistance, cardiovascular risk factors and those of diabetes mellitus. Evidence proves that changing the patients' life style by setting tiny goals gets clinically beneficial results even when the woman in question is still overweight or obese [7, 14, 27]. 

Practically all our patients had oligomenorrhoea or amenorrhoea at the beginning of the study, and it was remarkable to see that 6 of 16 patients in the Diamel treatment group were able to recover menstrual periods again, two from this group even managed to conceive, although one miscarried at 12 weeks. These results tie in with the evolution of the hormone biochemistry, as the LH is seen to decrease significantly in the group treated with Diamel; the testosterone gradually decreases too although when comparing the two groups it is only significant at the 3-month point in the treatment group. LH and high levels of insulin synergistically act to increase the production of androgens. Insulin resistance leads to hyperinsulinaemia, it reduces the SHBG, and it increases the free circulating testosterone, and together hyperandrogenism and hyperinsulinaemia alter the development of the follicles in the ovaries [2, 4, 8]. A favourable tendency is observed in our results with the Diamel since it seems that it helps reduce insulin resistance, LH, and testosterone levels. The possible utility of the Diamel would be in those women with PCOS that they suffer insulin resistance although other investigations with a greater reach as far as the study of sexual hormones would be necessary to justify their indication like therapeutic line in these patients. This syndrome is a chronic disease with symptoms that are suffered throughout a woman's life. It is a serious and common problem for public health that affects both the individual and the economy. A large percentage of women with PCOS also suffer from hyperandrogenism and insulin resistance, especially if they are overweight or if they are prone to the risk of metabolic syndrome, prediabetes, and type 2 diabetes. Tackling the problem must include providing support, education, and managing psychological factors, along with highlighting the importance of having a healthy life style and medical treatment. For a large majority of patients, the treatment involves a major change in their life style which is based on a multidisciplinary effort to prevent long-term complications. There must also be other alternative medicine available that do not have any adverse effects, and it would be completely acceptable to use Diamel in other investigations considering the results obtained.

In conclusion the food supplement Diamel reduces insulin resistance and improves insulin sensitivity in a small sample of women with PCOS and shows promise to be a nonmedical alternative if our findings are confirmed in larger randomized control trials.

## Figures and Tables

**Table 1:** Composition of Diamel (660 mg).

Arginine	35.5 mg	Glycine	7.1 mg
Ascorbic acid	10 mg	Ornithine	17.7 mg
Zinc sulphate	6 mg	Calcium pantothenate	1 mg
Folic acid	33 *μ*g	Cranberry extract	345 mg
Fumaric acid	35.5 mg	Lettuce extract	152 mg
L-carnitine	35.5 mg	L-cysteine	14.2 mg
Sodium methylparaben	0.33 mg	Pyridoxal	0.33 mg
Cyanocobalamin	0.16 mg		

**Table 2:** Clinic characteristics in women with PCOS.

Characteristics	Group A (Diamel) *n* = 18	Group B (placebo) *n* = 19
Actual age (years)	24.7 ± 4.6	28.0 ± 7.4
Diagnostic age (years)	19.1 ± 3.8	23.8 ± 5.4
Evolution (years)	7.7 ± 5.1	6.2 ± 5.3
Menarche age (years)	11.3 ± 1.4	12.5 ± 1.3
Weight (kg)	77.4 ± 13.2	82.4 ± 22.1
Height (cm)	159 ± 5.1	159.8 ± 7.4
BMI (kg/m^2^)	30.4 ± 5.1	31.4 ± 6.9

Values are means ± SD.

**Table 3:** Hormone and lipid values in patients with PCOS taken at the start of the clinical trial.

Measurements	Diamel	Placebo
FSH (UI/L)	6.7 ± 2.6	6.1 ± 1.5
LH (UI/L)	11 ± 6.1	9.7 ± 5.3
Testosterone (nmol/L)	2.9 ± 1.5	2.6 ± 1.6
Prolactin (mUI/L)	436.9 ± 130	434.1 ± 344
Cholesterol (nmol/L)	4.4 ± 0.7	4.5 ± 0.83
Triglycerides (nmol/L)	1.3 ± 0.6	1.6 ± 0.1
HDL-cholesterol (nmol/L)	1.03 ± 0.2	1.07 ± 0.3
LDL-cholesterol (nmol/L)	2.7 ± 0.7	3.2 ± 1.1

Values are mean ± SD.

**Table 4:** Evolution of weight and BMI in women with PCOS.

	Diamel	Placebo
Start	*n* = 18	*n* = 19
Weight (kg)	77.4 ± 13.2	82.4 ± 22.1
BMI (kg/m^2^)	30.4 ± 5.0	31.5 ± 6.9
At 3 months	*n* = 17	*n* = 16
Weight (kg)	76.4 ± 14	78.4 ± 21
BMI (kg/m^2^)	30.1 ± 5.2	30.4 ± 6.9
At 6 months	*n* = 16	*n* = 16
Weight (kg)	76.1 ± 15	78.2 ± 20
BMI (kg/m^2^)	29.9 ± 5.9	30.4 ± 6.8

Values are means ± SD.

**Table 5:** Evolution of the insulin resistance index (HOMA-IR) in patients with the polycystic ovary syndrome.

	*n*	Diamel	*n*	Placebo
HOMA-IR start	18	4.3± 2.4*	19	4.6 ± 2.5
HOMA-IR 3 months	17	2.6 ± 1.3	16	3.3 ± 2.0
HOMA-IR 6 months	16	2.4 ± 1.2*	16	3.3 ± 2.1

*P = 0.04.

Values are means ± SD.

**Table 6:** Evolution of the insulin sensitivity index (Raynaud) in patients with the polycystic ovary syndrome.

	*n*	Diamel	*n*	Placebo
Raynaud start	18	0.27 ± 0.1***	19	0.24 ± 0.1***
Raynaud 3 months	17	0.52 ± 0.3*	16	0.40 ± 0.2*
Raynaud 6 months	16	0.54 ± 0.3**	16	0.43 ± 0.24**

*P = 0.03.

**P = 0.005.

Values are means ± SD.

**Table 7:** Menstrual pattern in patients with polycystic ovary syndrome at the start and after the treatment.

Menstrual pattern	Diamel		Placebo	
	*n*	*n* (%)	*n*	*n* (%)
Oligomenorrhoea	18	17(94.4)	19	19 (100)
Amenorrhoea	18	9 (50)	19	8 (42.1)
Menstruation later treatment	16	6 (37.5)*	16	3 (18.6)*
Pregnancy	16	2 (12.5)	16	0

*P = 0.02.

**Table 8:** Evolution of the hormone levels in patients with PCOS during the follow up period.

Follow up	Groups	
	Diamel	Placebo
Start		
FSH (UI/L)	6.7 ± 2.6	6.1 ± 1.5
LH (UI/L)	11 ± 6.1*	9.7 ± 5.3
Testosterone (nmol/L)	2.9 ± 1.5	2.6 ± 1.6
3 months		
FSH (UI/L)	6.8 ± 2.3	5.8 ± 1.9
LH (UI/L)	9.9 ± 5.9	9.2 ± 5.4
Testosterone (nmol/L)	2.6 ± 1.3*	3.7 ± 3.2*
6 months		
FSH (UI/L)	5.8 ± 1.8	5.7 ± 1.9
LH (UI/L)	7.6 ± 4.9*	8.8 ± 4.2
Testosterone (nmol/L)	2.3 ± 1.6	2.4 ± 1.8

*P = 0.05.

Values are means ± SD.
